# The Male and Female Organs of Copulation in the Human Species and Some Mammalia, Examined and Demonstrated in Their Anatomical and Physiological Relations

**Published:** 1845-04

**Authors:** 


### ART. X.

*Die männlichen und weiblichen Wollust-Orgäne des Menschen und einiger Säugetheire in anatomisch-pathologischer Beziehung untersucht und dargestellt.* Von Dr. G. L. KOBELT, &c. Mit 5 Tafeln.——*Freiburg*, 1844.

*The Male and Female Organs of Copulation in the Human Species and some Mammalia, examined and demonstrated in their Anatomical and Physiological Relations.* By Dr. G. L. KOBELT, Prosector at Freiburg, &c. With 5 Plates.—*Freiburg*, 1844. 4to, pp. 66.

THIS is such a book as it does one's eyes good in these times to see. Tired with the microscope, and the long lines of chemical formulæ, they rest with thorough satisfaction on the large type of a full sized quarto full of old-fashioned anatomy. No thousandths of inches to calculate; no rows of naughts for decimals of French lines; but good old coarse and fine injections, and muscles in many layers, and fasciæ, visible nerves and arteries and great veins, and very moderate physiology; all that a prosector ought to know and be enthusiastic about—here it is, and the contrast with our last six months' reading is really quite delightful. The author needed not to apologize for the absence of any microscopic anatomy; the negation is one of the positive charms of his work.

We suspect our readers will be as glad as we were to find some new dissectional anatomy, and we shall therefore extract all we can, and arrange it not as Dr. Kobelt does, on a somewhat fantastic plan, but according to a system of anatomy: we will point out whatever is new to us in the several structures described in the anatomy of the copulative organs; omitting all that may be easily found elsewhere.

*Corpus cavernosum penis.* The crus penis is commonly said to arise on the inner lip of the ascending ramus of the ischium, and to unite with that of the opposite side *below* the arch of the pubes; but, when the parts are well injected, Dr. Kobelt says, that both the crura penis and its root lie, not below, but before the arch of the pubes; and as it were only applied against it, being fixed by their posterior parts to the sharp angle of the outer lip of the descending rami of the ossa pubis. Each crus enlarges before its union with that of the other side into a considerable bulb-like swelling, a true *bulbus corporis cavernosi penis,* which has been hitherto completely overlooked, probably, because it cannot be seen except in a well-injected penis, and is various in its degree of development. Just in front of these, at the part where the crura unite, the body of the penis is of comparatively small circumference; but beyond this contracted part it enlarges and remains of nearly equal size, to its connexion with the glans.

*Arteries of the corpus cavernosum.* These are not simply one to each side, as they are usually described; but, according to Kobelt, after the internal pudic artery has given off the artery of the bulb, and has divided into the A. dorsalis and the A. profunda penis, the last-named vessel (the artery of the corpus cavernosum of most writers) sends a branch half a line in diameter, from above downwards and within outwards, into the bulb of the crus penis, whose branches diverge widely directly after it has penetrated that part. One long branch goes backwards to the point of the crus penis; and another forwards into the body of the corpus cavernosum, where it anastomoses with the proper artery of the corpus cavernosum, which is the continuation of the A. profunda penis. The two A. profundæ penis anastomose at the root of the penis where the crura diverge, and form a short arch, from the convexity of which the A. corporis cavernosi of each side is given off.

*The veins of the corpus cavernosum* terminate in four or more sets. 1. Several, not commonly considered, ascend from the groove between the C. cavernosum and C. spongiosum, and winding round the sides of the former, end in the V. dorsalis penis. But of this set, also, some veins situated most posteriorly, pour their blood into the venous network by the side of the root of the penis, and not a few ascend to the cutaneous veins of the wall of the abdomen. 2. Numerous short branches from the interior of the C. cavernosum pass straightway through the fibrous covering, especially along the median groove, into the V. dorsalis. 3 Many pass in the angle between the crura penis into the prostatic and vesical venous plexus. And, 4, some pass through the inner surface of the crura penis and convey the blood partly into the internal pudic veins; and partly, by turning outwards, into the obturator veins.

*Bulb, glans, and corpus spongiosum urethræ.* It is only in his description of the first of these organs that we find Dr. Kobelt saying anything that is new as well as good. In its injected state, he says, the bulb of the C. spongiosum presents posteriorly two lateral hemispherical swellings, which are separated in the middle line by a longitudinal depression. This depression, of which an indication may be traced beyond the middle of the C. spongiosum urethræ, is caused by a septum which divides the posterior part of the bulb into two lateral symmetrical halves; but, anteriorly, is gradually lost. In the dog and stallion, the septum is very distinct: in man it can hardly be overlooked, and is noticed by Mr. Guthrie. The two portions of which it indicates that the bulb is composed, are the analogues of the completely divided portions of the bulb and C. spongiosum in the whole of the Marsupial tribe.

Moreover, each of these lateral "hemispheres" of the bulb is, in man, separated by a distinct furrow from a third superficial elevation (*colliculus bulbi intermedius*,) which is situated above and between them, but does not extend so far backwards as they do, and gives passage to the membranous part of the urethra, the vessels and nerves of the bulb, and the ducts of Cowper's glands.

There is, of course, no line of separation between the C. spongiosum of the urethra and that of the bulb: but neither does the erectile tissue terminate at the bulb; a portion (hitherto almost unnoticed,) is continued from it in a thin layer, which extends upwards and backwards, between the mucous and muscular layers of the whole circumference of the membranous part of the urethra, and is thence continued through the prostatic part into the neck of the bladder, between the coats of which it expands, its veins radiating and soon terminating in the external vesical veins. This portion of erectile tissue is especially developed in the caput gallinaginis, which derives from it all the properties of an erectile organ; and presents, when fully injected, all the characters of such an one. And it is this tissue, also, which is sometimes the seat of hemorrhoidal dilatations of veins. It would appear to serve to prevent the semen in emission from passing backwards into the bladder when, being turgid, the caput gallinaginis nearly fills the urethra; and it explains the difficulty or impossibility of discharging urine in the erect condition of the penis.

*Arteries of the bulb.* That which is commonly (or at least in the completest systems) described as a branch of the proper artery of the bulb going forward in the C. spongiosum urethræ to the glans, Kobelt says, is a separate branch of the pudic, in all cases except those in which the pudic divides into the dorsal and deep arteries of the penis before it gives off the artery of the bulb. When thus derived from the internal pudic, this artery (the bulbo-urethral) comes off about sixteen lines anterior to the artery of the bulb. It enters the penis at the angle between the crura, and after anastomosing with branches of the artery of the bulb, goes straight forwards in the upper wall of the C. spongiosum urethræ to the glans, where its branches anastomose with those of the A. dorsalis penis.

Thus, therefore, while the C. cavernosum penis is supplied with only one pair of arteries, the C. spongiosum, including the glans and bulb, receives three pairs, all of considerable size.

*Veins of the C. spongiosum, glans, and bulb.* The veins of the glans terminate in three sets. Those of the first set, passing over its posterior margin, form the commencement of the V. dorsalis penis; the second set run more deeply between the ends of the corpora cavernosa penis, and the hollow surface of the glans into which those ends fit, and, ascending between those parts, join the V. dorsalis; the third set is composed of veins which pass from the second set into the interior of the

C. cavernosa penis, and form communications between its veins and those of the glans.

The veins of the C. spongiosum urethræ are divided by Kobelt into four sets. 1. Those which pass out from the furrow on each side between the C. cavernosum and C. spongiosum, just behind the glans, and ascending obliquely backwards on the side of the C. cavernosum, join the V. dorsalis. 2. There is a plexus of veins, not hitherto described, but visible in well-injected specimens, between the upper surface of the C. spongiosum and the hollow in the C. cavernosum in which it lies. It is derived from branches which penetrate, in two nearly symmetrical rows, the upper wall of the C. spongiosum; from it the blood is carried by veins which ascend obliquely from the groove between the C. spongiosum and C. cavernosum on each side; and these veins, instead of passing like the similar veins of the first set into the V. dorsalis, pass to the side of the penis, where uniting with scrotal veins they form a network, from which the blood is conveyed to the inguinal cutaneous, the obturator, and the pudendal veins. 3. From the plexus just described on the upper surface of the C. spongiosum, veins pass, together with others which go directly upwards, from the C. spongiosum into the C. cavernosum, with the veins of which they form communications. 4. Veins passing out of the sides of the C. spongiosum also communicate freely with adjacent cutaneous veins.

The veins of the bulb are distributed in two modes. 1. Most of them pass from the back of the bulb under the pubic arch to the prostatic plexus. 2. Others from the *colliculus bulbi* (or third lobe) go outwards and backwards, and unite with the pudic veins.

*Muscles of the penis.* We should have thought that these two, if any, muscles had been already well enough described. But Dr. Kobelt says they have not, and that if the penis be first well injected any one may see more than he knew before. His account of them is as follows: First, of the *bulbo-cavernosus* or *accelerator urinæ.*

"It is divisible into three portions. Like the transversus perinei it may be separated into two layers. The fibres of the superficial layer arise from the well-known median tendinous line: they comprise two sets, which have the same general origin, and both proceed alike upwards and outwards, but at their insertions they diverge. 1. The fibres of the posterior three-fourths of this superficial layer pass upwards and outwards round the lower and lateral surfaces of the bulb, and pass (the posterior sooner than the anterior,) into a tendinous lamella, which turns itself in between the corresponding crus penis and the bulb, and over the upper part of the latter to unite with its fellow of the opposite side. This division, therefore, surrounds the bulb in the shape of a musculo-fibrous sheath, and will compress it from below upwards and from behind forwards: it may be called, *M. compressor bulbi proprius.*) The external sphincter ani and the transversus perinei superficialis, are connected with it in the middle line.

"2. The anterior fourth of the fibres of this superficial layer passes bridge-like over the groove between the C. cavernosum penis and the bulb; then the fibres wind forwards and upwards round the slightly constricted part of the penis which is seen (but hardly noticed) in this situation; and end in a fibrous layer, which is common to them, and the similar fibres of the opposite side, and covers the dorsal vessels and nerves. In this tendon (or fibrous layer) short muscular fibres are sometimes interwoven: Krause* has figured them, but ascribes them to the tendon of the ischio-cavernosus. The action of this division extends not only to the anterior part of the bulb, but, at the same time, to the body of the penis and its dorsal vessels and nerves: it is the true *M. bulbo-cavernosus*, or *M. constrictor radicis penis.*

* In Müller's Archiv, 1839, Heft i.

"3. The deeper layer consists, also, of two symmetrical lateral halves, but extends over only the posterior round portion of the bulb. The fibres arise from the median tendinous constriction of the bulb, extending backwards to the *colliculus bulbi.* The anterior fibres pass almost transversely outwards round the anterior border of the corresponding hemisphere of the bulb: the middle cover the convexity of the hemisphere: and the most posterior go almost straight forwards in the furrow between the hemisphere and the *colliculus;* and then, converging thus with the anterior fibres they are attached together to a small thin tendon which is connected with that of the opposite side in front of the situation at which the membranous part of the urethra dips into the bulb. The two muscles thus surround both the hemispheres in the form of a sling, or a closely applied muscular hood. They are separated from the superficial layer by cellular tissue in which nerves run, and are also distinguished by their direction and insertion. They might be considered distinct muscles, and named *M. compressor hemisperium* (i ?) *bulbi.* Their fibres (like those of the other layers) are transversely striated." (pp. 15-7.)

In explanation of the action of this muscle the author implies, that it serves to maintain and increase the turgescence of the glans penis, and thus to excite those nerves on which the peculiar sensation of the venereal orgasm depends. The turgescence is maintained both by retarding the reflux of the venous blood and by accelerating the onward course of that in the arteries. The retardation of the venous stream is easily and commonly accounted for by the pressure of the contracting muscle on the large veins; and this is accomplished according to the author's peculiar description of them, by the anterior portions of the muscles (M. constrictor radicis penis) compressing the V. dorsalis penis* under their common median tendon, at the same time that their upper margin (M.. compressor hemisphærii) compresses the veins of the bulb which emerge at the *colliculus.* The acceleration of the arterial current by the action of the same muscles is illustrated by a new and ingenious observation. On exposing the bulbo-cavernosi muscles in a dog just strangled or during strangulation, the author found that every time he irritated the glans penis (which, as well as the bulb, was always more or less turgid) the muscles suddenly twitched once or more, and forced the blood from the vessels of the bulb with a rapid jet through the vessels of the C. spongiosum urethræ, into those of the glans, which was thus, each time, made quite tense and glistening. Then, when the muscles ceased to act, the bulb again became turgid, and again might be compressed and partially emptied by fresh contractions of the muscles. Sometimes, a single irritation of the glans sufficed to produce a rhythmical succession of contractions of the bulbo-cavernosi muscles, so that the blood was forced from the alternately filled and emptied bulb, into the glans in a succession of jets which were like those produced by the alternating systole and dias tole of the heart. Similar compression of the bulb could be felt when the hand was pressed on the perineum of a dog during copulation: and in men, also, the contraction of the same muscles produces a similar increase of the turgescence of the glans. But in the strangulated dog no irritation of the penis which did not implicate the glans or the dorsal nerves produced any such effect.

From these facts the author deduces that the peculiar sensation of the venereal orgasm (Wollustgefühle,) is due to the sudden compression and

* Neither Müller nor Kobelt has ever found in man Dr. Houston's supposed M. compressor venæ dorsalis.

tension of the nerves which is thus produced, just as in congestions of the eye and ear subjective sensations of light and sound involuntarily impress themselves; and that "the two factors of this organico-physical process, the sensitive glans and the irritable muscular apparatus of the bulb, are related to each other as mutual and alternate excitors." (p. 20.)

Of course Dr. Kobelt denies to these muscles any such function as is implied in the name "accelerator urinæ," which they often bear. They have not even such an arrangement as could accelerate the flow of either urine or semen; and comparative anatomy shows that when the bulb is bifid, the corresponding muscles leave the urethra to invest the two divisions of the bulb, the compression of which is their sole function; and that, generally, their size is adapted not to the size of the urethra but to the size of the glans into which they have to pump the blood. The only real *accelerator* muscle in either man or other mammalia is, as Cuvier says, the compressor urethræ, the muscle surrounding the membranous part of the urethra.

In the last place (so far, at least, as the male sexual organs are concerned,) we shall quote the author's account of the ischio cavernosi muscles: which, like the last, he has examined especially when the penis has been distended, a measure which he considers almost necessary for displaying the exact extent and arrangement of the muscles.

"*The ischio-cavernosus muscle* corresponds on the whole to the general form of the crus penis, but surpasses it considerably in length. The middle or chief portion, arises between an inch and an inch and a half below the obtusely-rounded end of the crus penis, from the inner surface of the tuber ischii; it runs, first, straight upwards behind the ascending ramus, and then, increasing in size, turns under the ramus of the os pubis to pass to the crus penis, where it, for the most part, ends in the apex of a triangular tendinous lamella, which so covers the bulb-like enlargement of the penis that its basis lies in the constricted part of the penis near its root, and its lower angle at about the middle of the crus penis. Other muscular fibres come on the inner surface of the crus from the inner lip of the ramus of the os pubis, and go obliquely upwards and outwards to the inner margin of the tendinous lamella just mentioned. And a third portion of the muscle arises from the outer lip of the ramus of the os pubis, and goes upwards and forwards to be attached to the outer margin of the same lamella, or aponeurosis. Thus the M. ischio cavernosus is no band-like muscle, but a hollow or shell-shaped one which covers the whole free surface of the crus penis and its *bulb*." (pp. 29-30.)

In the flaccid state of the penis the ischio-cavernosus slightly retracts it; but

"During the artificial filling of the C. cavernosum [by injection] it expands itself over the whole convex surface of the crus and its bulb, as they expand and become rounded.......This process, we may also assume, takes place when the parts fill during life. When, now, the muscle, distended into a pouch-like form, presses both concentrically and longitudinally, the blood comprised within it must be pressed from it, so as to distend still more the already full body of the penis. But if the latter be incapable of receiving any further addition of blood, and thus the crus penis, and its bulb can empty themselves only imperfectly, or at last not at all, then have the C. cavernosum penis and the crura attained the highest degree of expansion and rigidity. Now, with every contraction of the two M. ischio-cavernosi, the external form of the penis will appear in its sharpest outlines, and it will assume its due position in regard to the pelvis." (pp. 33-4.)

These muscles, like the bulbo-cavernosi, were seen to contract in the strangled dog every time the glans, provided it was turgid, was stimulated. And a similar contraction, when the penis is more or less erect, may be felt in the human perineum; so that the completest filling of the erectile tissue, produced by the coincident *pumping* contractions of the M. ischio- and bulbo-cavernosi will ensue on each successive irritation of the glans, producing coincident reflex actions of these muscles. And their coincident action is necessary; because if one pair alone acted, they might press the blood from one portion of the erectile tissue into the other (e. g. from the C. spongiosum urethræ into the C. cavernosum penis, if the M. bulbo-cavernosi alone contracted), and so neither portion would be made turgid.

Such is our author's account of the male organs of copulation, and he must be admitted to have made in it a handsome contribution to our knowledge of anatomy. We shall next follow him, more briefly, in his description of the corresponding organs of the female.

That which he chiefly has in view is to show the exact analogy existing between certain of the copulative organs in the male and female; an analogy which has been commonly allowed as a general fact, but has not yet been strictly and particularly demonstrated. We shall here follow the arrangement of the author more nearly than in the preceding division of the subject. The principal analogues of the male organs which he describes are the female organs corresponding to the glans, C. spongiosum urethræ, bulb, and bulbo-cavernosus muscle.

The analogue of the glans penis is, of course, the *glans* clitoridis. Its form, the author says, corresponds exactly to that of the glans penis; and the peculiarities which, in different species, the latter organ presents are always, in the same species, exactly imitated in it: in those species, also, of which the males have an os penis, as the otters, hares, cats, &c., the clitoris of the females has a similar portion of bone or cartilage in it.

The nerves of the glans clitoridis are even much more abundant than those of the glans penis: the two dorsal nerves of the former are, in proportion, three or four times larger than those of the latter. Each of the nerves runs, usually divided in three branches, to the margin of the glans, and in its substance they form intricate plexuses; but their size, where they penetrate, is such, that one might wonder where they can find room to be distributed in so small and so vascular an organ. They have the same relation to the constrictor Cunni muscles as the corresponding nerves of the glans penis have to the bulbo-cavernosi.

The author next demonstrates, as the analogue of the male C. spongiosum urethræ, a venous plexus, which he names (not very instructively) *pars intermedia*: and here we had better remind the reader that the erectile tissue of the C. spongiosum urethræ has no essential relation to the urethræ as a *urinary*, but only as a *seminal* canal, and that therefore an organ may be analogous to it without having any connexion with the urinary apparatus, provided it has corresponding relations to the analogues of the glans and bulb. As then the C. spongiosum urethræ is a *pars intermedia* between the glans penis and bulb, so is the organ or part now to be described a *pars intermedia* between the glans clitoridis and that which, in the female, corresponds to the bulb.

The *pars intermedia*, then, is a venous plexus formed of variously convoluted, but chiefly longitudinal veins, which lies, on each side, under the body of the clitoris, and tends outwards, diverging from it and becoming broader. Its upper and smaller part is formed by veins, which may be divided into three sets; namely, 1, some which form lateral branches of the V. dorsalis clitoridis; 2, those which penetrate the lower surface of the body of the clitoris, and are exactly analogous to the communicating veins between the C. spongiosum urethræ and the C. cavernosum penis; 3, others which come to it from below, from the frenulum, nymphæ, and labia, and which correspond to those described as the fourth set of veins of the C. spongiosum penis.

The arteries of this *pars intermedia* are branches of the internal pudic, which correspond to the bulbo-urethral arteries of the male.

By their relative position between the glans clitoridis and the organs next to be described—their venous spongy texture, the origin and destination of their veins, and the origin of their arteries,—the author thinks that it is demonstrated that these organs are the analogue of the C. spongiosum urethræ. J. F. Meckel and Autenrieth considered the nymphæ to be the analogues of the C. spongiosum; but Kobelt has repeatedly injected them without detecting a trace of cavernous texture in them. We think he has fully established his point; and his evidence is strengthened by what we have next to say of the analogue of the bulb.

The *Bulbus Vestibuli* has an interesting history given in detail by the author, and of which the outlines may be traced by the synonymes he gives it; namely, *plexus retiformis*, R. de Graaf;* *crura clitoridis interna*, Swammerdam;† *plexus cavernosus*, Tabarranus;‡ *corpus cavernosum*, Santorini;§ *bulbe du vagin*, of the French; semi-bulb, Taylor,|| &c. It is an immediate continuation and extension of the posterior parts of the two *pars intermediæ.* It is therefore composed of two portions, one of which is placed on each side, and has the shape of a full leech. It (i. e. the portion on each side) is attached to the ramus of the pubic arch with its larger bluntly rounded (tail) end directed downwards and backwards; its smaller (head) end lying above, under the root of the clitoris; its convex (back) surface resting on the ramus; and its concave (abdominal) surface turned to the cavity of the vagina; so that the two together occupy the space between the beginning or vestibule of the vagina and the rami of the pubic arch. When injected it is, on an average, an inch and a quarter long, six or seven lines broad, and from four to six lines thick. In the uninjected state it is like an almond-shaped swelling, of a dark blueish-red colour, and giving out, when cut, a half-coagulated viscid bloody fluid, like that from the bulb of the male. It is composed of a close-packed plexus of intricately anastomosing veins, which have a general longitudinal direction, and of which the mass is inclosed in a fibrous investment.

From numerous injections the author gives this account of the veins of the *female bulb.* They are divisible into four sets. 1. Branches of communication, at the anterior small end of the bulb, with the veins of the *pars intermedia.* 2. Branches of communication at the same part, between the bulbs of opposite sides, or rather between the two divisions of the bulb. 3. Those from the posterior margin of the upper part, which

* De mulierum organis. † Miraculum naturæ seu uteri muliebris fabrica.

‡ Observationes anatomicæ, p. 65. § Observationes anatomicæ, § iii, p. 207.

|| Dublin Journal of Medical Science, vol. xiii, p. 104.

pass to a very dense and delicate venous plexus in and upon the upper wall of the vagina, and in the neck and even the walls of the bladder. These correspond to the continuation of the spongy tissue of the male bulb along the membranous and prostatic portions of the male urethra, as already described. 4. And lastly, the proper efferent veins of the bulb (the analogues of the proper *venæ bulbosæ* of the male,) which proceed from the lower and larger end of the bulb, and empty their blood, partly into the pudic, and partly into the external hemorrhoidal veins.

The arteries of the female bulb are arranged like those of the male; but so little is commonly said about the distribution of the internal pudic artery in the female, that we shall give the author's account of it complete.

"After the common pudic artery has given off the transversalis perinei, it divides into two nearly equal branches, one of which (A. labialis posterior,) passes round the lower end of the bulb and goes to the labium and nympha. The other branch, or properly the continuation of the pudic runs along the posterior margin of the bulb upwards towards the root of the clitoris. In this course it gives off—

"1. A tolerably large branch into the posterior round part of the lower end of the bulb (arteria bulbosa), noticed hitherto by Theile alone.

"2. A branch of uncertain size to the anterior wall of the urinary bladder (A. vesicalis anterior; constant but not hitherto described.) At this point the artery divides into—

"3. The A. dorsalis clitoridis; and

"4. The A. profunda clitoridis. And this, then, gives off—

"5. A small vessel which passes through and under the crus clitoridis to the pars intermedia......and

"6. A small branch in the crus clitoridis. Lastly, its continuation forms with the corresponding vessel of the opposite side, behind the bifurcation of the clitoris—

"7. An anastomotic arch; from which there proceeds on each side—

"8. A small branch which sinks into the corpus cavernosum." (p. 46.)

Some have regarded this bulb as analogous to the C. spongiosum urethræ, (as Krause and Theile;) some have compared it with the male bulb, (as Louth, Taylor, Malgaigne;) but they have not shown distinctly the grounds of comparison. Dr. Kobelt, adopting the latter opinion, gives the particulars of the analogy thus briefly:

"Its bulbous form, its internal texture, its immediate parenchymatous continuation with the representative of the C. spongiosum urethræ (the *pars intermedia*), its connexion with the spongy tissue of the membranous part of the urethra and the neck of the bladder, its afferent and efferent blood-vessels, its nerves, its relation to Cowper's gland, and, lastly, the circumstance of its being covered by a compressing muscle, agree in so multiform and yet so complete a manner with all the similar relations and characters of the male bulb, that we must regard it as the equivalent of that organ. The division into two lateral halves cannot disturb this conclusion; this is to be regarded only as a persistence in an earlier type, which is observed at one period in the male embryo as well, and of which the traces are to be recognized in even the developed male bulb, in the duplicity of its hemispheres, its muscles, vessels, and nerves, as well as in its permanent (partial) septum, &c. Nay, we have seen above that a partial division of the male bulb is constant in many animals, and a complete division of it in the marsupialia throughout life. If one imagines the male bulb divided, and its halves diverging to the sides and backwards, they would have to be looked for in no other place than that in which the corresponding half-organs in the female are found. And, on the other hand, if the two bulbs of the female be pressed together towards the middle line, we should have, in respect of position and form, a complete male bulb, with a septum consisting of two layers, a fibrous sheath, a muscular covering, &c." (pp. 47-8.)

It is by the arrangement of this muscular covering that the analogy is completed; for the author follows Sabatier, Winslow, and many others in regarding the M. constrictor Cunni (as it has been erroneously called,) as the analogue, both in anatomy and in function, of the male M. bulbo-carvernosus. His description of it differs from those of nearly all his predecessors, except the singularly accurate Santorini, and its accuracy may be ascribed to the advantage acquired for dissecting it, by previously injecting the veins and putting it on the stretch. He says:

"It is always a double muscle. It arises with a broad expanded base from the perineal fascia, about midway between the orifice of the anus and the tuber ischii. Its fibres are here often widely spread out, so that the inner ones sometimes mingle with those of the sphincter ani, or the outer ones are in contact with the ascending ramus of the ischium. From this origin it proceeds, gradually becoming smaller and converging with that of the opposite side, forwards and upwards towards the clitoris, and covers or rather surrounds in this course the *bulbus vestibuli* in its whole length and breadth. .... It consists of two flat portions, of which the posterior with its band-like tendon slips through between the upper and posterior margin of the bulb and the clitoris, and unites with the corresponding portion of the opposite muscle in a common broad tendinous layer which is placed over the cavernous venous tissue of the urethra. The anterior flat portion ascends, on the contrary, to the dorsum of the clitoris and unites with that of the opposite side in a flat thin tendon under which the dorsal vessels and nerves pass. Not unfrequently there is, by the side of the clitoris, a small piece of muscle, woven into this tendon, and corresponding to that represented by Krause in the penis." (pp. 48-9.)

Here we conclude, for the author's physiology is, as we have said, not so good as his anatomy; and his account of the applications of the organs which he has described is a little too vivid for these pages. If our notice of his work should meet his eye, he may be assured that he will be regarded by many of his brethren in England as among the best of demonstrator-anatomists, a thoroughly good observer, and a very worthy pupil of the veteran Tiedemann.

---

